# Evoked oscillatory cortical activity during acute pain: Probing brain in pain by transcranial magnetic stimulation combined with electroencephalogram

**DOI:** 10.1002/hbm.26679

**Published:** 2024-04-15

**Authors:** Enrico De Martino, Adenauer Casali, Silvia Casarotto, Gabriel Hassan, Bruno Andry Couto, Mario Rosanova, Thomas Graven-Nielsen, Daniel Ciampi de Andrade

**Affiliations:** 1 Center for Neuroplasticity and Pain (CNAP), Department of Health Science and Technology, Faculty of Medicine, Aalborg University, Aalborg, Denmark; 2 Institute of Science and Technology, Federal University of São Paulo, São Paulo, Brazil; 3 Department of Biomedical and Clinical Sciences, University of Milan, Milan, Italy; 4 IRCCS Fondazione Don Carlo Gnocchi, Milan, Italy

**Keywords:** acute pain, alpha, electroencephalogram, event-related spectral perturbation, inter-trial coherence, transcranial magnetic stimulation

## Abstract

Temporal dynamics of local cortical rhythms during acute pain remain largely unknown. The current study used a novel approach based on transcranial magnetic stimulation combined with electroencephalogram (TMS-EEG) to investigate evoked-oscillatory cortical activity during acute pain. Motor (M1) and dorsolateral prefrontal cortex (DLPFC) were probed by TMS, respectively, to record oscillatory power (event-related spectral perturbation and relative spectral power) and phase synchronization (inter-trial coherence) by 63 EEG channels during experimentally induced acute heat pain in 24 healthy participants. TMS-EEG was recorded before, during, and after noxious heat (acute pain condition) and non-noxious warm (Control condition), delivered in a randomized sequence. The main frequency bands (α, β1, and β2) of TMS-evoked potentials after M1 and DLPFC stimulation were recorded close to the TMS coil and remotely. Cold and heat pain thresholds were measured before TMS-EEG. Over M1, acute pain decreased α-band oscillatory power locally and α-band phase synchronization remotely in parietal–occipital clusters compared with non-noxious warm (all *p* < .05). The remote (parietal–occipital) decrease in α-band phase synchronization during acute pain correlated with the cold (*p* = .001) and heat pain thresholds (*p* = .023) and to local (M1) α-band oscillatory power decrease (*p* = .024). Over DLPFC, acute pain only decreased β1-band power locally compared with non-noxious warm (*p* = .015). Thus, evoked-oscillatory cortical activity to M1 stimulation is reduced by acute pain in central and parietal–occipital regions and correlated with pain sensitivity, in contrast to DLPFC, which had only local effects. This finding expands the significance of α and β band oscillations and may have relevance for pain therapies.

## 1 Introduction

Along with structural connections, neuronal assemblies exchange information through oscillatory activity (Buzsáki & Draguhn, 2004). Neuronal oscillations and their synchronization, concomitantly occurring in different frequency bands, enable information processing across spatially distant brain regions by inter-areal phase-locking. This process creates time windows when information can be integrated concomitantly in sparse neuronal clusters, allowing complexity to emerge (Fries, 2015). Increasing evidence indicates that individuals with acute and chronic pain exhibit altered electroencephalogram (EEG; Ploner et al., 2017). During acute heat pain, the activation of cortical networks resulted in modifications in different frequency bands, including α-band (Furman et al., 2018), β-band (Nickel et al., 2020), and γ-band (Schulz et al., 2015) in healthy individuals. In chronic pain patients, decreased high α-band and low β-band (10–20 Hz) were reported to occur, coupled with an increase in high β-band (20–30 Hz; Mussigmann et al., 2022), and related to pain symptoms in peripheral neuropathic pain (Teixeira et al., 2021). While changes in the β-band tend to be confined to frontal cortical regions, changes in the α-band appeared to be more widespread but rather located in central and parietal–occipital cortical regions (De Martino et al., 2021; Furman et al., 2018; Mussigmann et al., 2022). Slowing of the peak of the α rhythm over the sensorimotor cortex has also been suggested as a possible and reliable biomarker of pain sensitivity (Furman et al., 2020). However, our understanding of brain oscillations during pain states is mainly based on studies investigating resting-state EEG, not being designed to provide detailed insights into the alterations in oscillatory dynamics of specific cortical regions.

Evoked oscillatory dynamics of a specific brain region can be assessed by recording the EEG responses after perturbating pulses of transcranial magnetic stimulation (TMS-EEG). TMS-EEG measurements can be performed by targeting the primary motor cortex (M1), which is known to have major connections to interoceptive and cognitive networks (Buzsáki & Draguhn, 2004), but also targeting extramotor areas, such as the dorsolateral prefrontal cortex (DLPFC; Kähkönen et al., 2005). A key feature of TMS-EEG is the ability to transiently affect both the oscillatory power (i.e., event-related spectral perturbation) and phase synchronization (i.e., inter-trial coherence) in the targeted cortical region (Casali et al., 2010). While oscillatory power refers to the magnitude of brain oscillations in a specific frequency band and is related to cortical excitability, phase synchronization refers to phase coherence of TMS-evoked potential (TEP) responses over multiple trials (Makeig et al., 2004). To date, it is unknown how pain influences the evoked-oscillatory cortical activity of cortical areas highly relevant to pain cortical networks, such as M1 (Gordon et al., 2023) and DLPFC (Seminowicz, 2017). Unveiling these patterns would provide unique and novel models of how specific cortical areas react to pain stimuli locally and how they drive and engage responses in remote areas, which could have major mechanistic and therapeutic implications.

Here, we used TMS-EEG to probe cortical oscillations in M1 and DLPFC during induced acute heat pain and nonpainful warm control stimulation in healthy participants. Based on previous studies showing changes in α-band and β-band (Furman et al., 2018; Furman et al., 2020; Nickel et al., 2020), we hypothesized that acute heat pain would affect oscillations in the α-band at central regions and remotely in parietal–occipital regions when targeting M1, whereas DLPFC stimulation would affect oscillations in the β-band in the frontal region.

## 2 Methods

### 2.1 Participants

In adherence to the Helsinki Declaration, the study was approved by the local ethics committee (Videnskabsetiske Komite for Region Nord-jylland: N-20220018) and was registered at ClinicalTrials.gov (NCT05566444). This study is based on original data from a study in which the cortical excitability results have been reported, measured through local peak-to-peak amplitude and slope, and global mean field power (De Martino et al., 2023). A total of 24 healthy (12 females) right-handed individuals were included (age: 27 ± 5.5 years, weight: 70 ± 14 kg, height: 173 ± 10 cm). All participants were healthy, did not suffer from neuropsychological or other medical conditions, and did not assume any medicaments. Sample size calculations were determined based on previous data exclusively related to local and global cortical excitability results to provide 80% power, type I error rate of 0.05, and type II error rate of 20% (De Martino et al., 2023).

### 2.2 Experimental protocol

**Figure 1:**
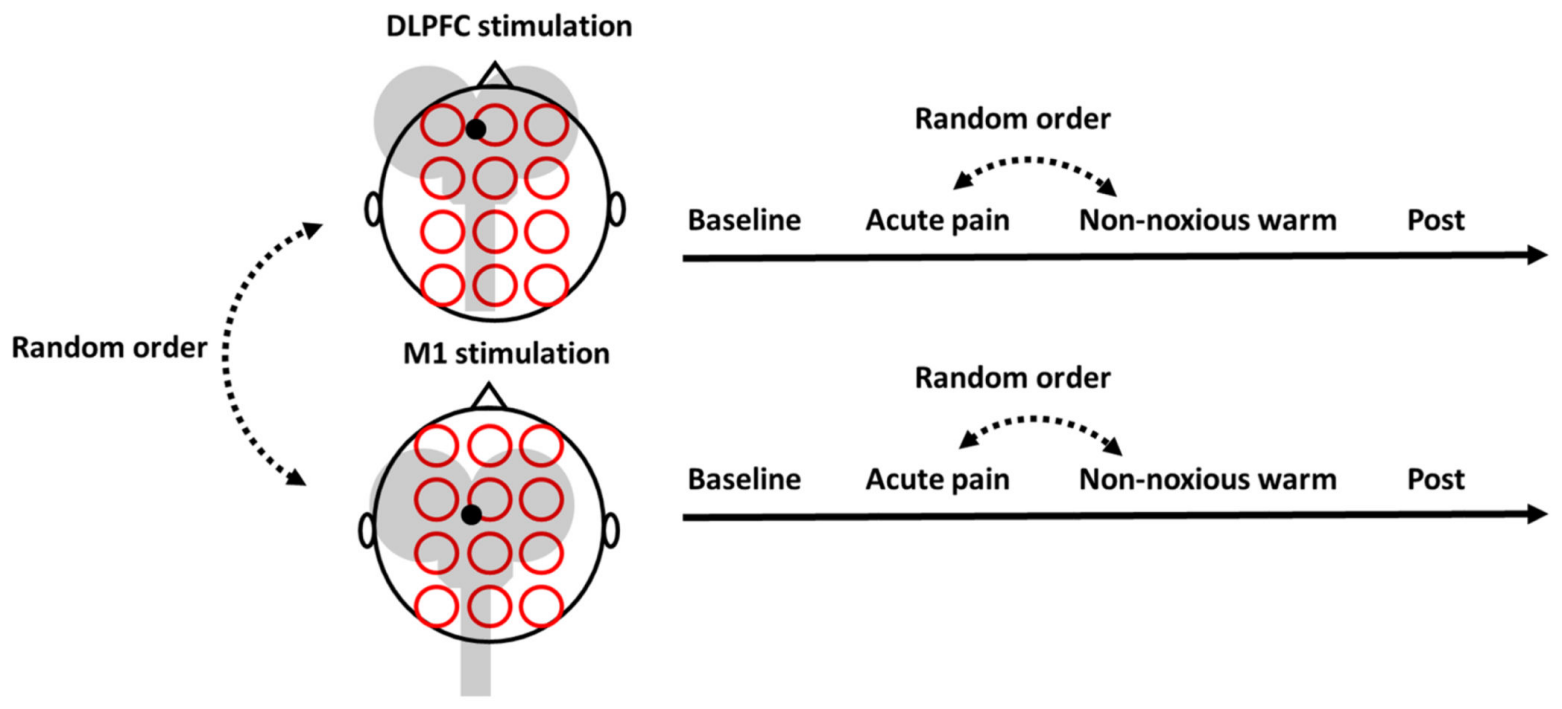
Transcranial magnetic stimulation-electroencephalography was performed in two cortical regions: the dorsolateral prefrontal cortex (DLPFC) and primary motor cortex (M1). Four different conditions were collected for each cortical area: baseline, acute pain, non-noxious warm, and post.

In a single experimental session, eight TMS-EEG blocks were performed by probing two cortical areas relevant for pain modulation (left DLPFC and left M1) under four distinct conditions: baseline, acute pain, non-noxious warm, and post (Figure 1). The sequence of cortical stimulation areas and the order of acute pain and non-noxious warm conditions were randomized among participants to ensure equal distribution and balance between the two groups in terms of participant number. A 5-min interval separated each block, and a 30-min break was provided between cortical areas. The duration of each TMS-EEG block was approximately 7 min. Pain thresholds for each participant were determined using a thermode (Medoc advanced medical system, Haifa, Israel) with a Peltier-based probe (3 × 3 cm) placed on the right forearm’s volar region. Starting at 32°C, the warm detection threshold (WDT) was determined by the methods of limits. WDT was measured by an increasing temperature (1°C/s) until the participant perceived warm and pressed a stop button. Heat- and cold-pain thresholds (HPT and CPT) were measured similarly by asking the participant to press a stop button when the pain was perceived (average of 3 runs) (Rolke et al., 2006), and approximately 30 s separated each measurement. Heat- and cold-pain thresholds were chosen because previous studies showed the effects of repetitive TMS on DLPFC and M1 on these outcomes (Ciampi De Andrade et al., 2014; De Martino et al., 2019; Moisset et al., 2015; Taylor et al., 2012). Before experiments, the temperature to be used in the acute pain and non-noxious warm stimulation sessions was determined. For acute pain and non-noxious warm stimulation, participants indicated the probe temperature needed to produce moderately intense heat pain (acute pain) and a harmless warm sensation (non-noxious warm). Beginning at HPT, the probe temperature increased in 1°C increments until participants reported moderately intense heat pain, rated as 5 out of 10 on a numerical scale (0 being no pain, 10 being the worst pain imaginable). Temperatures of 45.2 ± 0.7°C were used for acute pain conditions during TMS-EEG data collection, while 40.2 ± 0.8°C (below HPT) induced the innocuous warm sensation (non-noxious warm). To ensure consistency in pain intensity during stimulation, participants were asked to rate the intensity of pain at the end of each recording session, as previously described (Schulz et al., 2015). For baseline and post measurements, a 32°C thermode stimulator probe (skin temperature) was used to prevent any thermal sensation. Additional information can be found in De Martino et al. (2023).

### 2.3 Electroencephalographic recordings during transcranial magnetic stimulation

A biphasic stimulator (Magstim Super Rapid^2^ Plus^1^, Magstim Co. Ltd, Dyfed, United Kingdom) and a figure-eight-shaped coil (70 mm, Double Air Film Coil) were used to stimulate DLPFC and M1. TMS-evoked potentials (TEP) were recorded using a TMS-compatible passive electrode cap with 64 electrodes (EASYCAP GmbH, Etterschlag, Germany) placed according to the 10–5 system, with the Cz electrode aligned to the vertex of the head. The electrode impendence was maintained below 5 kΩ during the recordings. Raw signals were amplified and sampled at 4800 Hz (g.HIamp EEG amplifier, g.tec-medical engineering GmbH, Schiedlberg, Austria). The online reference was on a forehead electrode, two electrodes recorded the electrooculogram (EOG) on the lateral side of the eyes, and the ground electrode was situated midway between the eyebrows. To reduce neck and shoulder postural muscle activity, participants sat on an ergonomic chair equipped with dedicated neck support. To minimize oculomotor muscle activity, participants had an easy-to-see fixation spot on the wall. To abolish auditory responses to TMS coil clicks, TMS-click sound masking toolbox (TAAC) with noise-canceling in-ear headphones (Shure SE215-CL-E Sound Isolating, Shure Incorporated, United States) was used (Russo et al., 2022). Finally, to reduce somatosensory sensations from the TMS coil and any EEG electrode movement artefacts, two net caps (GVB-geliMED GmbH, Ginsterweg Bad Segeberg, Germany) with a plastic stretch wrap handle film were applied over the EEG cap.

Using an optical-tracking system, a navigated brain stimulation system (Brainsight TMS Neuronavigation, Rogue Research Inc., Montréal, Canada) calibrated the head of the participant and TMS coil position. The optical-tracking system also generated a 3D brain reconstruction using template MRI (Brainsight software, Rogue Research), scaling to the head of the participant to optimize the reliability of targeting during sessions. The M1 target was identified near the left hemisphere’s hand knob of the central sulcus, where the largest motor-evoked potential (MEP) was recorded by electromyographic electrodes placed on the right first dorsal interosseous muscle (FDI) (i.e., the hand “hot spot”). The resting motor threshold (rMT) was determined as the TMS intensity required to produce MEPs greater than 50 μV in 5 out of 10 trials, with pulses delivered at 0.2 Hz, as measured from the FDI muscle electromyography (Rossini et al., 2015). Disposable surface silver/silver chloride adhesive electrodes (Ambu Neuroline 720, Ballerup, Denmark) were used to record MEPs in the FDI muscle, placed parallel to the muscle fibers. A reference electrode was positioned on the ulnar styloid process. To avoid sensory feedback contamination, M1 TMS-evoked potentials were applied below the rMT (90% of rMT) (Fecchio et al., 2017). The DLPFC target was identified in the middle frontal gyrus following Mylius et al. (2013), with the stimulator intensity set at 110% of rMT of the FDI muscle. A real-time visualization tool (rt-TEP) was used to ensure detectable TMS-evoked potentials in both cortical targets (Casarotto et al., 2022), allowing minor adjustments of TMS coil orientation across participants to reduce variance and guarantee a minimum of 6 μV in the early peak-to-peak amplitude response in the average of 20 trials in the nearest EEG electrode to DLPFC and M1 targets. The navigated brain stimulation system and rt-TEP were utilized throughout the study to monitor TMS coil location (within 3 mm of cortical targets) and the highest signal-to-noise ratio in EEG recordings. For each condition (baseline, acute pain, non-noxious warm, and post), approximately 160–180 pulses (~8 min of TMS stimulation) were administered, with interstimulus intervals randomly jittered between 2600 and 3400 ms to prevent significant reorganization/plasticity processes from interfering with longitudinal TMS/EEG measurements (Casarotto et al., 2010). At the end of the experimental session, a sham TMS coil (70 mm, Sham Double Air Film Coil) was applied with the same intensity and location as the active TMS coil to confirm the absence of substantial early (below 100 ms) evoked responses.

### 2.4 Data processing

Data preprocessing was carried out using customized algorithms based on the EEGlab toolbox (Delorme & Makeig, 2004) running on Matlab R2019b (The MathWorks, Inc., Natick, MA). EEG signals were divided into trials of 1600 ms around the TMS pulse (±800 ms with time 0 corresponding to the TMS pulse). The TMS artifact was removed from all EEG recordings by replacing the recording between −2 and 6 ms from the TMS pulse with prepulse signal (−11 to −3 ms). Epochs and channels containing noise, eye blinks, eye movements, or muscle artifacts were visually inspected, cataloged, and discarded. Epochs were band-pass filtered (2–80 Hz, Butterworth, third order) and down-sampled to 1200 Hz. Channels were re-referenced to the average reference, baseline corrected, and the four conditions (baseline, acute pain, non-noxious warm, and post) were concatenated. In this merged dataset, independent component analysis using the EEGLAB runica function was employed to eliminate any residual artifacts, including eye blinks, lateral eye movements, heartbeats, muscle tonic contractions of facial and neck muscles, and residual of the TMS artifacts. Subsequently, epochs were re-segmented within a ±600 ms time window, and the combined dataset was divided back into the original four conditions. Spherical splines were used to interpolate bad channels (Delorme & Makeig, 2004).

The following TMS-evoked EEG parameters were extracted in the time-frequency domain:

1. The event-related spectral perturbation (ERSP) and relative spectral power (RSP) were calculated to quantify the power amplitude independent of phase. The ERSP allows identifying the changes in power as a function of time and frequency, and RSP provides specific normalized information about the distribution of power across frequency bands.
2. Inter-trial coherence (ITC) was extracted as a measure of phase synchronization.

Time-frequency maps were extracted between 8 and 45 Hz using Morlet wavelets with 3.5 cycles as implemented in the EEGLAB tool-box and previously reported (Donati et al., 2021; Ferrarelli et al., 2012; Rosanova et al., 2009). ERSP was computed from the time-frequency maps as the ratio of the spectral power of individual EEG trials relative to the pre-stimulus period (Donati et al., 2023; Rosanova et al., 2009). The significance of ERSP maps with respect to the baseline was assessed by bootstrapping samples from the prestimulus period (500 permutations, two-sided comparison, *p*-value < .05 after false discovery rate (FDR) correction for multiple comparisons). Mean power spectra were then calculated by averaging significant ERSP values across channels and time samples. RSP was extracted from the mean power spectra as the percentage of power in a given frequency band (Donati et al., 2021). Finally, ITC was calculated by normalizing the complex-valued single-trial time-frequency values by their corresponding moduli and taking the absolute value of the across-trials averaged results. The significance of ITC maps with respect to the baseline was assessed by bootstrapping samples from the pre-stimulus period (500 permutations, one-sided *p*-value < .05 after FDR), and significant ITC values were averaged across channels, time samples, and frequency bands.

**Figure 2:**
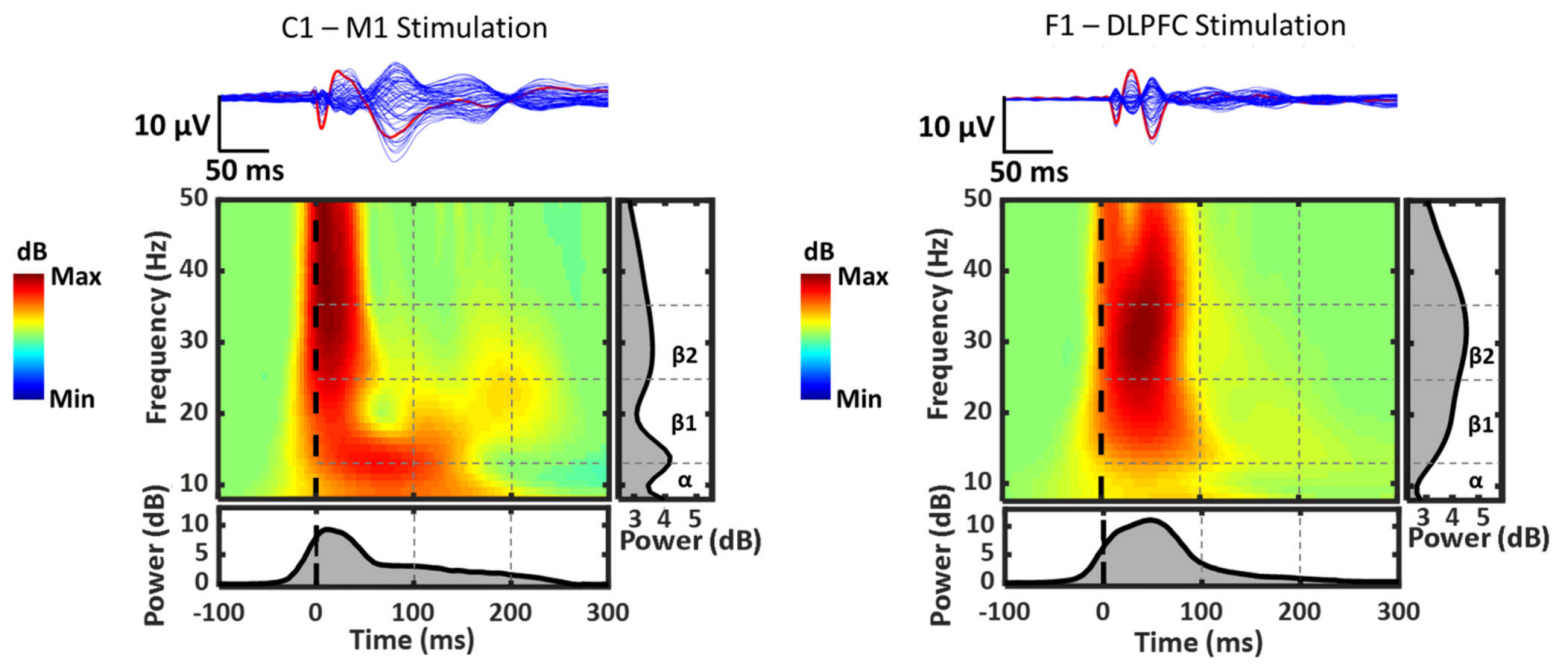
Sample data of transcranial magnetic stimulation (TMS)-evoked potentials recorded with EEG following single pulse stimulation in a representative participant. The figures depict the primary motor cortex (M1) and dorsolateral prefrontal cortex (DLPFC) stimulation with the butterfly plot and topographical maps. The red line corresponds to the C1 and F1 electrodes, and the blue lines correspond to the other 62 channels. The bottom panel shows significant ERSP maps calculated on C1 and F1 electrodes. The grayscale graph plotted at the right depicts the mean power spectrum profile in the α, β1, and β2 bands during the 300 ms after TMS, and the grayscale graph plotted below depicts the mean broadband evoked power during time.

The EEG channels were organized into clusters by averaging individual channels to determine regional neural activity, and ITC, ERSP, and RSP were extracted over a time interval of 6–300 ms after the TMS stimulus for four distinct frequency bands: α (8–12 Hz), β1 (13–24 Hz) and β2 (25–34 Hz). For the statistical analysis, the absolute change from baseline was calculated for acute pain, non-noxious warm, and post. Evoked responses were assessed locally where the TMS pulse was delivered (Figure 2) and remotely in related cortical regions, that is, those areas typically presenting reverberating oscillations within the natural frequencies of the TMS target. Thus, local responses for M1 probing were assessed in the α-band on the middle centro-frontal cluster (Table 1), while for DLPFC stimulation, local responses were assessed in the β1-band and β2-band on the left/middle/right prefrontal clusters (Table 2). Remote effects of M1 probing were also assessed where α-band activity has its natural peak frequency [i.e., posterior-occipital regions (De Martino et al., 2021; Sarnthein et al., 2006) situated in left/middle/right parieto-occipital clusters; Table 1]. Remote prefrontal cluster responses were assessed to control for the expected α-preponderant parietal–occipital cluster responses triggered by M1 probing, while remote responses in parietal–occipital areas were assessed after DLPFC perturbations to control for the expected frontal β-band responses.

If frequency bands and clusters revealed significant differences between acute pain and its comparators in the broader 6–300 time interval, then differences in shorter time intervals were explored (i.e., 6–100, 100–200, and 200–300 ms) so that it would be possible to determine whether the significant changes occurred early, middle, or later relative to the probing TMS pulse.

Importantly, reduced α power during acute experimental pain has been reported in resting state-EEG experiments (Chang et al., 2002, 2003). Therefore, we planned a supplementary assessment to rule out a potential confounding pain-related decrease in α power before the delivery of the probing TMS pulse (time interval −600 ms to −10 ms). The α power was calculated by applying the Fast-Fourier Transform to the spontaneous EEG of the pre-TMS stimulus for each individual trial and then averaging the resulting power spectrum across trials. This analysis was conducted exclusively in the electrode clusters exhibiting statistical differences between acute pain and other conditions.

### 2.5 Statistical analysis

Statistical analysis was performed with the Statistical Package for Social Sciences (SPSS, version 25; IBM, Chicago, IL). Results were presented as means and standard deviation with a two-sided 5% significance level set for statistical significance and if not otherwise stated. All data from thermal stimulation (acute pain and non-noxious warm) and for post-measures are reported as absolute changes from the baseline. All measurements were assessed by visually examining histograms and Shapiro–Wilk tests. Due to several non-normally distributed parameters, Friedman tests were used to analyze ESRP, RSP, and ITC for each band frequency and cluster, as well as for the α power before TMS stimulation. Post hoc analyses were performed with Wilcoxon’s multiple comparison tests, and Bonferroni correction was applied when necessary. To determine whether functional connectivity changes during acute pain were associated with HPT and CPT, Spearman’s rank correlation analyses were conducted between HTP and CPT and the absolute changes from the baseline of the ERSP, RSP, and ITC during acute pain. Only significant changes in specific frequency bands and clusters were considered for correlations. Finally, Spearman’s rank correlation analysis was performed on the absolute changes from the baseline between α-ERSP and α-ITC from M1 stimulation to investigate whether local α changes correlated with remote α changes since both were significantly changed during acute pain.

## 3 Results

All volunteers participated in all experimental sessions and underwent all assessments. No adverse events related to TMS-EEG or thermal stimulations were present.

### 3.1 Spectral power changes

**Figure 3:**
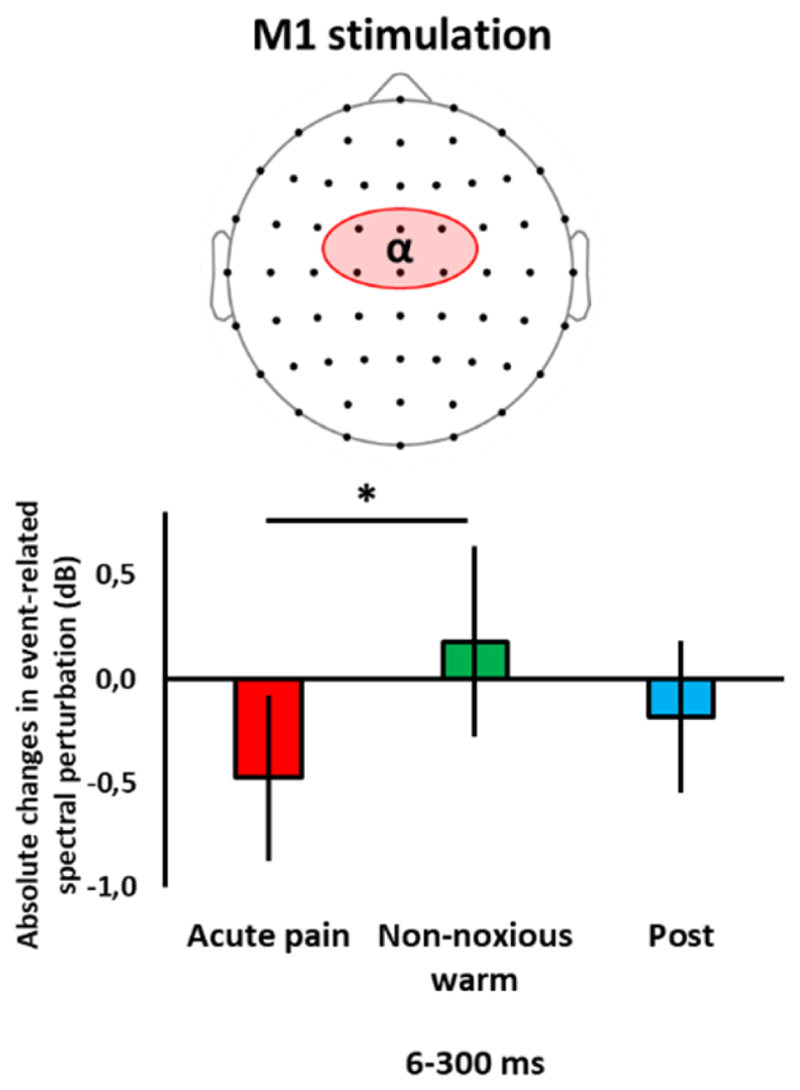
The event-related spectral perturbation absolute changes from baseline (mean and 95% confidence interval) in the middle centro-frontal cluster are shown during acute pain, non-noxious warm, and post (Wilcoxon test * *p* < .05 - Bonferroni corrected).

**Figure 4:**
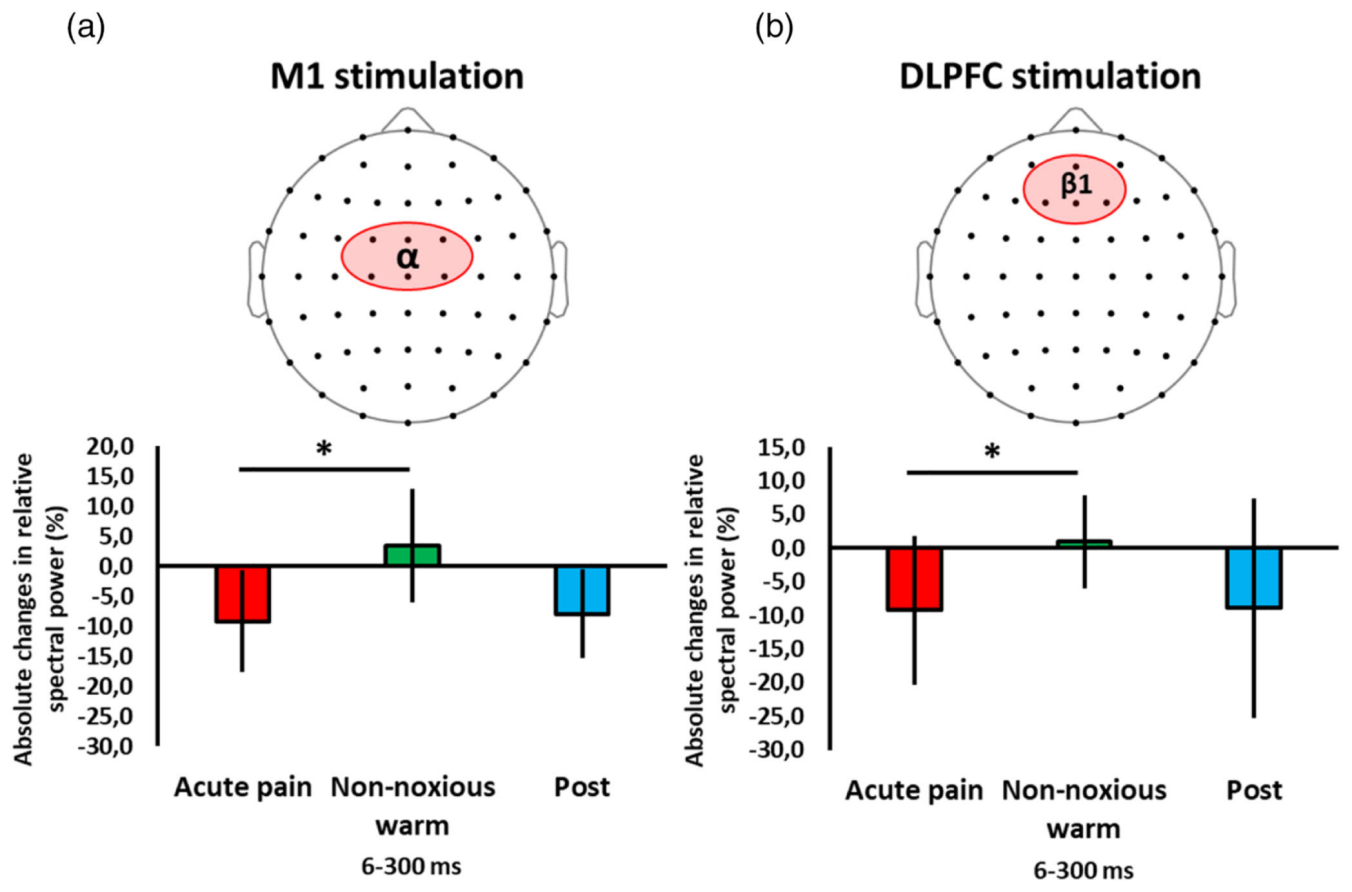
The relative spectral power absolute changes from baseline (mean and 95% confidence interval) in the middle prefrontal cluster are shown during acute pain, non-noxious warm, and post (Wilcoxon test * *p* < .05 – Bonferroni corrected).

Upon M1 probing, a significant decrease in the power of α-band ERSP was found locally in the middle centro-frontal cluster (Chi-square = 10.083; *p* = .006) as well as in the α-band RSP (Chisquare = 10.750; *p* = .005) at the time interval 6–300 ms (non-normalized parameters are reported in Table S1). Post hoc analysis revealed a significant decrease during acute pain compared to non-noxious warm in α-band ESRP (*p* = .003; Bonferroni-corrected; Figure 3) and in α-band RSP (*p* = .009; Bonferroni-corrected; Figure 4a). Shorter time intervals were then explored for changes in α-band ERSP after M1 probing in the middle centro-frontal cluster. Significant differences were found at the time interval 6–100 ms (Chi-square = 6.750; *p* = .034), 100–200 ms (Chisquare = 11.083; *p* = .004), and 200–300 ms (Chi-square = 10.333; *p* = .006) so that for all three-time intervals post hoc analysis revealed a significant decrease in acute pain compared to non-noxious warm (6–100 ms, *p* = .003; 100–200 ms, *p* = .006, 200–300, *p* = .012; all Bonferroni-corrected; Figure S1). A significant difference was also detected in later latencies for α-band RSP (200–300 ms: Chisquare = 7.583; *p* = .023), but post hoc analysis did not detect any difference between conditions (*p* > .05; Bonferroni-corrected).

Upon DLPFC probing, a reduction in power in the β1-band RSP was found locally (middle prefrontal cluster; Chi-square = 12.250; *p* = .002) at the time interval 6–300 ms. Post hoc analysis revealed a decrease in acute pain compared to non-noxious warm (*p* = .015; Bonferroni-corrected; Figure 4b). When shorter time intervals were analyzed for the β1-band RSP, a difference was found to be only localized at the time interval 6–100 ms (Chi-square: 9.250; *p* = .010). Post hoc analysis revealed a reduction in RSP in the β1-band power during acute pain compared to non-noxious warm (*p* = .018; Bonferroni-corrected; Figure S2). No local differences were detected in the ERSP (all *p* > .05). Probing of the DLPFC did not lead to significant ERSP and RSP changes in the α-band, β1-band, and β2-band on the left and right prefrontal clusters (all *p* > .05—non-normalized parameters are reported in Table S2).

### 3.2 Absence of confounding pre-probing alpha power changes

The preplanned sensitivity analyses confirmed that the decrease in α-band ERSP described above was not significantly present before the TMS in the middle centro-frontal cluster. The spontaneous pre-TMS pulse α-band power was 0.88 ± 0.62 dB for baseline, 0.94 ± 0.62 dB for acute pain, 0.94 ± 0.63 dB for non-noxious warm, and 0.90 ± 0.60 dB for post. These differences were not statistically different (Chi-square = 0.583; *p* = .747).

### 3.3 Phase synchronization changes

**Figure 5:**
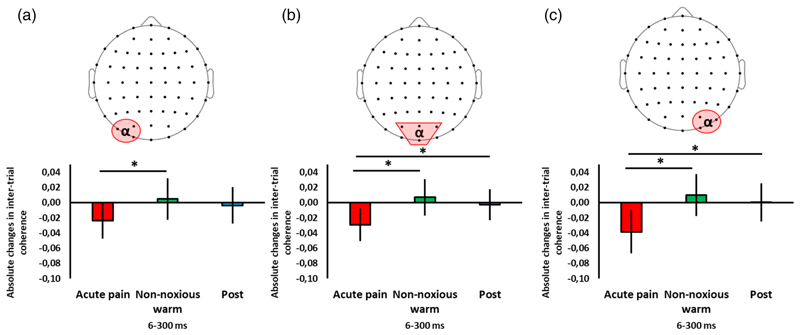
The inter-trial coherence absolute changes from baseline (mean and 95% confidence interval) in the left (a), middle (b), and right (c) parieto-occipital clusters are shown during acute pain, non-noxious warm, and post (Wilcoxon test * *p* < .05 - Bonferroni corrected).

Upon M1 probing, a significant reduction was found in ITC in the α-band locally at the time interval of 6–300 ms. Remote reductions in ITC were significant in parieto-occipital regions: the right (Chi-square = 12.250; *p* = .002), middle (Chi-square = 13.583; *p* = .001), and left parieto-occipital (Chi-square = 6.750; *p* = .034) clusters. Post hoc analysis confirmed a reduction in α-band ITC after acute pain compared to non-noxious warm in all three EEG clusters (right parieto-occipital cluster, *p* = .006; middle parieto-occipital cluster, *p* = .003, and left parieto-occipital cluster, *p* = .021; all Bonferroni-corrected) and between acute pain and postcondition within the right (*p* = .009; Bonferroni-corrected) and middle (*p* = .012; Bonferroni-corrected) parieto-occipital clusters (Figure 5). The reductions in α-band ITC occurred in both short and middle latencies intervals in the right parieto-occipital channel clusters. For the right parieto-occipital cluster, we found a reduction in the following time intervals: 6–100 ms (Chi-square = 11.516; *p* = .003) and 100–200 ms (Chi-square = 9.979; *p* = .007). In both two-time intervals, post hoc analysis revealed a decrease in acute pain compared to non-noxious warm (6–100 ms, *p* = .009; 100–200 ms, *p* = .006; all Bonferroni-corrected) and in acute pain compared to postcondition (6–100 ms, *p* = .006; 100–200 ms, *p* = .012; all Bonferroni-corrected; Figure S3). Within the left and middle parietal–occipital clusters, the α-band ITC showed significant decreases in acute pain compared to non-noxious warm in the later time interval (200–300 ms). No change was found in the local middle centro-frontal clusters (Chi-square = 0.750; *p* = .687; non-normalized parameters are reported in Table S3).

Probing of the DLPFC did not lead to significant ITC changes in the α-band, β1-band, and β2-band on the left, middle, and right prefrontal clusters (all *p* > .05—non-normalized parameters are reported in Table S4).

### 3.4 Correlations

**Figure 6:**
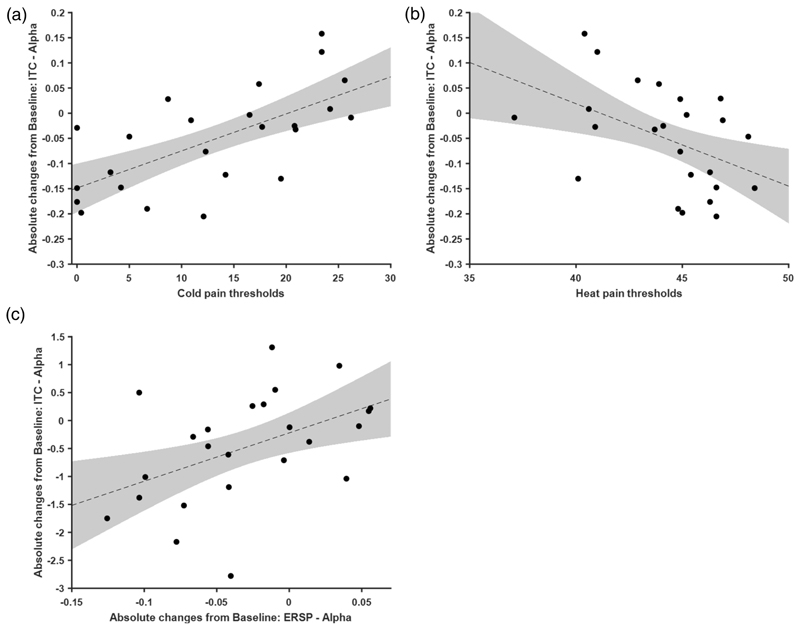
Correlation of the α inter-trial coherence (ITC) responses within the right parieto-occipital cluster from M1 stimulation during acute pain, expressed as absolute changes from baseline. This correlation was measured against cold (a) and heat pain thresholds (b) in the time interval of 6–100 ms. (c) Correlation of the α ITC responses within the middle parieto-occipital cluster from M1 stimulation during acute pain, expressed as absolute changes from baseline. This correlation was measured against α event-related spectral perturbation (ERSP) within the middle centro-frontal cluster in the time interval of 6–100 ms.

During acute pain, reduction in α-band ITC significantly correlated with cold (rho = .638, *p* = .001; Figure 6a) and heat pain thresholds (rho = −.463, *p* = .023; Figure 6b) in earlier latencies (6–100 ms) after M1 stimulation. Upon M1 probing, reduction in α-band ITC locally under acute pain significantly correlated with the decreases in α-band ERSP in earlier latencies (rho = .459, *p* = .024; Figure 6c). Upon DLPFC probing, no correlations were found between thermal thresholds or local reductions in β1-band power during acute pain.

## 4 Discussion

The current study investigated the effects of acute pain on evoked oscillatory cortical activity by applying single-pulse TMS to two distinct cortical regions. In terms of power dynamics, acute pain resulted in power reductions localized at clusters near the respective stimulation targets and within specific frequency bands: we observed local decreases within the α-band under M1 stimulation and within the β1-band during DLPFC probing. In terms of phase dynamics, acute pain led to a decrease in α-band synchronization in parietal–occipital clusters under M1 stimulation. Notably, these changes in α-band ITC correlated with thermal pain thresholds, suggesting a potential interaction between trait pain perception and dynamic acute pain-related disengagement of posterior areas, probably via corticothalamic loops.

Traditional resting-state EEG has demonstrated power amplitude and peak frequency changes across various bands in chronic pain patients (Mussigmann et al., 2022) and healthy participants during experimental pain (Nickel et al., 2017). In the presence of neuropathic pain, enhanced *θ* and high-*β* power were described, as well as a decrease in the high α and low-*β* power (Sarnthein et al., 2006). Furthermore, a shift toward lower peak α frequency was also described in patients affected by neuropathic pain patients (de Vries et al., 2013). Studies using tonic painful heat stimuli reported a decrease in peak α frequency oscillations in parietal regions (Nir et al., 2010), a decrease in α and β oscillations in the central region (Nickel et al., 2017; Peng et al., 2014; Wang et al., 2023), while faster frequency oscillations power in the middle prefrontal cortex were seen to increase (Schulz et al., 2015). While these results have offered valuable insights into pain mechanisms, resting-state EEG has limited capability in probing the excitability and connectivity of specific cortical circuits. In the current study, a different methodological approach has been applied, taking advantage of TMS-evoked EEG oscillations, which reflect both local and remote activations from connected populations with various electrophysiological properties as well as the reactivity of the neuronal population at the stimulation site (Massimini et al., 2005; Rosanova et al., 2009). The key finding of the present study was that the alteration in power dynamics during acute pain depends on the cortical region engaged by the TMS stimulation. M1 and DLPFC are part of two different structural and functional connectivity arrangements and are hubs in different brain networks (Menon, 2013). Dissimilarities between M1 and DLPFC have been described in the spatial–temporal dynamics of M1 activity propagations after TMS stimulation to M1: after the engagement of the stimulation target, activity spreads to more parietal locations via corticocortical volleys from M1 to S1, and to the opposite hemisphere, via the corpus callosum (Komssi et al., 2002). Differently, the left DLPFC TMS has been described to activate the local stimulation area, as well as the opposite prefrontal cortex (Komssi et al., 2004). Accordingly, the present results showed that acute pain entrained frequency-specific changes in power depending on the network being probed. The fact that acute pain caused a reduction in power in the α-band after M1 and β-band after DLPFC stimulations can be interpreted according to the natural frequency framework (Rosanova et al., 2009). This means that the main oscillatory frequency evoked by a probing pulse of TMS would be the dominant frequency naturally occurring on that specific cortical area at rest, being the α-band for sensorimotor (Mu rhythm) and β-band for premotor ones. The reduction in power on both targets during acute pain would be in line with previous data showing decreased corticospinal excitability during acute pain (Burns et al., 2016). It is possible that local increases in thresholds (i.e., lower excitability) would allow these regions to disengage from their current motor/cognitive processing, thus allowing for plasticity-driven reorganizational changes necessary to respond to acute pain. This aligns with corticospinal excitability modifications in patients with chronic pain, where motor thresholds are rarely abnormal, and plastic changes are more frequently related to intracortical GABA and glutamate-dependent changes (Mhalla et al., 2010).

Another main finding of the present study was the reduction of posterior α-band synchronization during acute pain when M1 was probed. While α-band ESRP was locally reduced at the stimulation site in the motor region, decreased α-band ITC did not occur locally. Instead, it took place over remote parietal–occipital electrodes. These changes were more pronounced at early latencies (<100 ms) after the M1 pulse and contralaterally. It has been shown that the thalamus acts as the primary pacemaker for α oscillations, with the pulvinar (Saalmann et al., 2012) and lateral geniculate nucleus (Hughes et al., 2011) preferentially driving the α rhythm. However, other studies indicate that α waves propagate from higher- to lower-order areas in both the sensorimotor and posterior cortices (from the associative cortex towards the primary cortex) and then to the thalamus, likely via short-range supragranular feedback projections (Halgren et al., 2019). Intracranial recordings have shown that cortical pyramidal cells modulate excitability and create synchronized feedback loops to the thalamus, leading to highly coherent oscillations (Halassa & Sherman, 2019) and supporting the idea that sensorimotor and posterior cortices play a role in initiating and coordinating oscillations generated within the thalamus (Destexhe et al., 1999). This process involves cortex–thalamus–cortex loops, potentially explaining the generation of large-scale coherent oscillations within the thalamocortical system (Fuggetta et al., 2005). We found that M1 stimulation during acute pain decreases the expected parieto-occipital phase synchronization of ongoing rhythmic activity. The intensity of this effect correlated with both the heat and cold pain thresholds of participants, which is one of the few correlations between connectivity metrics and individual trait nociceptive thresholds reported to date. These findings suggest that during acute pain, M1 engages less intensely distant phase synchronization (i.e., lower ITC) in those healthy participants with “trait” higher pain thresholds (i.e., broader non-noxious temperature limen between cold and heat pain thresholds). Therefore, individuals with lower pain sensitivity traits (i.e., higher thermal pain thresholds) exhibit lower inhibitory effects of acute pain in α-band ITC. These correlations reached moderate strength for shorter latencies. Analogously, in the visual system, the accurate perception of the temporal sequence of visual events depends on the phases of the α rhythm (Mathewson et al., 2009). It was also known that sensorimotor networks oscillate at 10–20 Hz (Jensen et al., 2005), which are the frequencies shown to reduce pain intensity in repetitive TMS trials targeting M1 (Halassa & Sherman, 2019), and which is according to Hebbian models (Scarpetta et al., 2002).

There are limitations that should be considered when interpreting these findings. First, this study did not evaluate the saliency of the non-noxious warm stimulus, which could influence the results. The non-noxious warm condition served as a control to provide comparable sensory inputs to the forearm without inducing pain. We administered non-noxious warm stimuli to control for attentional effects on the forearm. We believe that the further qualitative differences between acute thermal pain and acute thermal non-noxious pain stimuli are intrinsic to the experience of pain, which is inherently more intense and attention-grabbing than non-painful stimulus. In this study, we did not intend to dissect the individual role of attention and salience in the experience of acute pain under TMS-EEG. However, it is important to acknowledge that acute heat pain and warm non-noxious stimuli may engage saliency systems differently. While it has been argued that heightened salience is an intrinsic component of pain, a control situation with matched salience intensity delivering a different sensory stimulus could be potentially useful. Second, although the current study focused on two major cortical targets, namely M1 and DLPFC, pain engages numerous other cortical regions not probed here. Future research should explore targets like the parietal cortex or deep cortical areas like the posterior insula cortex, which are essential in pain processing (Dongyang et al., 2021). Expanding the cortical targets examined using TMS-EEG can give a more comprehensive understanding of pain mechanisms. Furthermore, utilizing multiple corrections might inadvertently neglect potentially significant findings and incur type-II errors (Rothman, 1990). Third, source modeling analysis was not applied in the current study, which could have enabled a more fine-grained localization of cortical areas involved in pain-related neural activity. In the current study, individual MRI scans were not available for target localization, potentially affecting the precision, but not reliability of anatomical targeting. Indeed, our findings indicate distinct evoked responses in two stimulation areas, suggesting effective targeting of different cortical regions. Specifically, stimulation of the primary motor cortex consistently elicited prominent responses in electrodes C3 and C1, while stimulation of the DLPFC resulted in significant responses in electrodes F1 and F3. Additionally, the observed differences in natural frequencies—mainly alpha/low beta activity in M1 and high beta activity in DLPFC—support the notion that distinct cortical areas were targeted. Cortical responses induced by TMS can be affected by auditory and somato-sensory responses (Belardinelli et al., 2019). To mitigate this, a control condition with the non-noxious warm stimulus was included, and compared differences based on changes from baseline and post-stimulation phases. Additionally, we analyzed short-time intervals as auditory and somatosensory responses predominantly influence the 100–200 ms range (Rocchi et al., 2021) despite implementing measures to minimize their impact (Casarotto et al., 2022; Russo et al., 2022). Additionally, the neural gamma oscillation was not analyzed in the current study because the frontalis or temporalis muscle recorded from the scalp has spectral features that resemble gamma activity (Chouchou et al., 2021).

In conclusion, our results indicated that TMS stimulations to M1 during acute pain drive frequency-specific remote phase synchronization effects, which correlated with nociceptive thresholds and were qualitatively and quantitatively different from the responses seen after DLPFC probing. Our findings likely expand the significance of α-band and *β*-band oscillations in perceptual processes to now include nociception. These results are relevant to understanding the M1 and DLPFC neuroplastic effects induced by pain states, and future studies should investigate the effects of rTMS targeting M1 and DLPFC to determine whether these treatments can modulate these frequency bands during pain states on an individual basis [see Ciampi de Andrade and García-Larrea (2023), for review].

## Supplementary Material

Supplementary material

## Figures and Tables

**Table 1:** EEG clusters along with their corresponding electrodes used for M1 stimulation.

Cluster name	Electrodes
Middle centro-frontal	FCz, Cz, FC1, FC2, C1, C2
Middle parieto-occipital	POz, Oz, PO3, PO4
Left parieto-occipital	PO7, PO3, O1
Right parieto-occipital	PO8, PO4, O2

**Table 2:** EEG clusters along with their corresponding electrodes used for DLPFC stimulation.

Cluster name	Electrodes
Middle prefrontal	AFz, Fz, F1, F2
Left prefrontal	AF7, AF3, F5, F3
Right prefrontal	AF8, AF4, F6, F4

## Data Availability

The data that support the findings of this study are available from the corresponding author upon reasonable request.

## Practitioner Points

1. Acute pain resulted in α-band power reduction localized at clusters near the respective stimulation targets under M1 stimulation.
2. Acute pain resulted in β1-band power reduction localized at clusters near the respective stimulation targets under DLPFC stimulation.
3. Acute pain led to a decrease in α-band synchronization in parietal-occipital clusters under M1 stimulation.
